# Bird and mammal species composition in distinct geographic regions and their relationships with environmental factors across multiple spatial scales

**DOI:** 10.1002/ece3.1072

**Published:** 2014-04-21

**Authors:** Rafi Kent, Avi Bar-Massada, Yohay Carmel

**Affiliations:** 1 Department of Plant Sciences, University of CambridgeDowning Street, Cambridge, CB23EA, U.K; 2 Department of Civil and Environmental Engineering, The Technion – Israel Institute of TechnologyHaifa, 32000, Israel; 3 Department of Biology and Environment, University of Haifa at OranimKiryat Tivon, 36006, Israel

**Keywords:** Canonical correspondence analysis, environmental determinants, multiple scales, occurrence data

## Abstract

Global patters of species distributions and their underlying mechanisms are a major question in ecology, and the need for multi-scale analyses has been recognized. Previous studies recognized climate, topography, habitat heterogeneity and disturbance as important variables affecting such patterns. Here we report on analyses of species composition – environment relationships among different taxonomic groups in two continents, and the components of such relationships, in the contiguous USA and Australia. We used partial Canonical Correspondence Analysis of occurrence records of mammals and breeding birds from the Global Biodiversity Information Facility, to quantify relationships between species composition and environmental variables in remote geographic regions at multiple spatial scales, with extents ranging from 10^5^ to 10^7^ km^2^ and sampling grids from 10 to 10,000 km^2^. We evaluated the concept that two elements contribute to the impact of environmental variables on composition: the strength of species' affinity to an environmental variable, and the amount of variance in the variable. To disentangle these two elements, we analyzed correlations between resulting trends and the amount of variance contained in different environmental variables to isolate the mechanisms behind the observed relationships. We found that climate and land use-land cover are responsible for most explained variance in species composition, regardless of scale, taxonomic group and geographic region. However, the amount of variance in species composition attributed to land use / land cover (LULC) was closely related to the amount of intrinsic variability in LULC in the USA, but not in Australia, while the effect of climate on species composition was negatively correlated to the variability found in the climatic variables. The low variance in climate, compared to LULC, suggests that species in both taxonomic groups have strong affinity to climate, thus it has a strong effect on species distribution and community composition, while the opposite is true for LULC.

## Introduction

Many studies have attempted to find common patterns in the global and regional distribution patterns of different taxa, and furthermore, common processes governing those distributions (e.g., Pianka 1966; Whittaker et al. 1973; Gaston 2000; Hubbell 2001; Allen et al. 2002; Kent et al. 2011; Keil et al. 2012). The distributions of birds and mammals, among many other taxonomic groups, and their relationships with their environment have been studied extensively. Species distributions have generally been attributed to one or more environmental variables or variable groups, such as topography (e.g., Whittaker 1956; Terborgh 1971; Kavanagh et al. 1995; Melo et al. 2009); climate and water availability (e.g., Whittaker 1956; Bohning-Gaese 1997; Lennon et al. 2000; Steinitz et al. 2006; Kent et al. 2011; Keil et al. 2012); habitat characteristics such as heterogeneity (Bohning-Gaese 1997; Veech and Crist 2007), disturbance (Kavanagh et al. 1995), and land-use and land-cover (LULC) type (Kent et al. 2011; Keil et al. 2012).

The relationships between species composition and the environment depend on spatial scale (Levin 2000; Kent and Carmel 2011; Kent et al. 2011). It is now accepted that analyses of relationships between species composition and the environment should be conducted at multiple spatial scales in order not to overlook important factors (Kent et al. 2011; Dray et al. 2012; Blank and Carmel 2013). Some environmental variables, specifically those with slow rates of change in time and space (e.g., annual temperature and precipitation), are expected to affect species composition at coarse scales. Other faster changing variables, such as topographic relief and soil type, may affect local patterns of biodiversity (Keil et al. 2012).

Corroborating this prediction, we found in a recent study that mammal species composition in the contiguous USA corresponded strongly to climate and land-use–land-cover variables at all spatial scales of the analysis (grains from 4.5 to ∼100 km and extents from 20,500 km^2^ to the entire contiguous USA, Kent et al. 2011). Land use was more important than climate at fine scales, while climate became the prevalent factor at coarser scales (namely, for extents broader than 2.6 × 10^6^ km^2^).

The effects of environmental variables on species composition may be related to their location along the environmental gradient (Steinitz et al. 2006). A similar environmental difference has a larger effect closer to the edges of the gradient (e.g., an increase in precipitation in arid environments is more influential than a similar increase in a mesic ecosystem). Extending this concept, we propose that two elements determine the strength of the observed effect of an environmental factor on species composition independently: (1) its ecological affinity and (2) its variability. A given environmental variable may be important if its ecological affinity is high, even if its variability is low. Similarly, high variability may render an environmental factor effective, even if its direct ecological impact is relatively weak. One goal of this paper is to evaluate the feasibility of this proposition in the context of environmental determinants of species composition. In order to disentangle these two elements, we record the coefficient of variation (CV) for each environmental factor at each scale. We then relate the variability in species composition explained by a given factor, to its specific CV. We expect that across scales, CV of environmental factors is positively related to the impact of this factor on species composition.

The second goal of this paper is a general evaluation of the relationships between species composition and the environment, extending the analysis of mammals in USA (Kent et al. 2011). Toward this end, we carried out extended analyses on two taxonomic groups (mammals and breeding birds) in two distant continents (the contiguous USA and Australia). An interesting question in this respect is the importance of continent-specific factors versus the importance of taxon-specific biology. The continent shape and position across latitudes determine the range and distribution of climates. Similarly, the spatial patterns of LULC are different between continents. These differences may strongly affect the relations between environmental factors and species composition across scales. If this is true, or if mammals/birds in Australia are ecologically very different from mammals/birds in the USA, then results for mammals in the USA may be very different from those for mammals in Australia. In contrast, one may hypothesize that taxon-specific biological traits may be more important for the relationships between species composition and environmental factors. If this is correct, then we may expect to find high similarity between trends of mammals in both continents and between trends of birds in both continents, but not between birds and mammals in the same continent.

## Materials and Methods

We used recorded occurrences of bird and mammal species in the USA and Australia, obtained from the Global Biodiversity Information Facility (GBIF) using the GBIF database from November 2011 (GBIF 2008). In order to control the geographic accuracy of the records used in the analyses, we only used records with three or more decimal digits in both longitude and latitude coordinates. Collection and museum data, as well as data providing portals such as GBIF, include several types of bias, originating from different sampling errors (Guralnick et al. 2007; Kadmon et al. 2009; Kent and Carmel 2011). A rigorous testing of the effect of inherent biases in GBIF data (Kent and Carmel 2011) concluded that under strict use of multiple species, and across large spatial extents, biases in the data have no significant effect on species composition analyses using multivariate analyses, like the one we conducted here. Several recent studies also concluded that occurrence records may be used for ecological spatial studies under certain constrictions (Graham et al. 2007; Loiselle et al. 2008), offsetting the inherent biases in collection data. An additional measure that can be taken to reduce the effect of taxonomic bias in GBIF data is the use of external taxonomic lists (Guralnick et al. 2007). Here, we filtered our data using only bird species that breed in the respective regions, using a species list from the Breeding Bird Survey of North America and a similar list provided by an expert ornithologist from Australia (J. Szabo, pers. comm.). We omitted all bat species from the mammal datasets, as we assumed that their ecological requirements are very different from those of terrestrial mammals.

In addition to occurrence records, we compiled GIS layers of environmental variables from the two regions, using remotely sensed data (LULC from http://glcf.umiacs.umd.edu/ and NDVI from http://www.fao.org/geonetwork), and fine scale global climatic and elevation variables available from WorldClim (Hijmans et al. 2005). All environmental variables (Table 1) were available at a spatial resolution of 0.00833^0^ longitude (equivalent to ∼1 km^2^ around the equator) or finer. In order to record the values of the different variables at various grain sizes, we resampled the variable layers using the mean value for continuous variables and majority value for categorical variables. Scale was altered quantitatively by simultaneously altering both grain and extent, in order to maximize the explanatory power of environmental variables simultaneously (for details, see Appendix). Spatial scale, as defined here, consists of two components following Wiens (1989). Extent is the area covered by a delineation of all sampling locations in a given study area. Each extent consists of a basic sampling grid. The size of a single cell in a given sampling grid is the grain size. When moving up from the finest scale to the next coarser scale, we doubled the length of the side of each grid cell. We repeated this process 10 times. We created an ArcGIS python script that generated sets of square sampling grids of extent *E* and grain *g* at each scale (Table 2). In each sampling grid, comprising 32 × 32 pixels (total 1024 cells), the script returned the number of pixels with species occurrences and the number of species in the grid. In order to meet the requirements of multivariate analyses, we set a threshold on the amount of data in each of the sampling grids. A sampling grid was included in the analyses if it met two conditions. First, it had to include at least 30 grid cells with nonsingleton occurrences (more than one occurrence record per cell), and second, it contained data on at least six different species. For each selected sampling grid that complied with the thresholds, and at each scale, we ran a partial Canonical Correspondence Analysis (pCCA) using the vegan package (Oksanen et al. 2008) in the R statistical software package, version 2.12 (R Core Team 2010). For pCCA, we divided the environmental variables into four groups: climate (mean annual temperature, temperature seasonality, mean annual precipitation, and precipitation seasonality), topography (elevation and elevation range), land use–land cover (distance to urban areas, population density, and percentages of agriculture, forest, grasslands, urban, surface waters, and wetland areas), and NDVI. We then applied pCCA to each group separately (ter Braak 1986; ter Braak and Verdonschot 1995; Cushman and Mcgarigal 2002; Legendre et al. 2005). To calculate the amount of variance in species composition explained by each variable and each group, we divided the inertia of each group in each sampling grid by the overall inertia in the respective sampling grid and multiplied it by 100. Total inertia is an expression of the amount of variance in the species data within the sampling grids (ter Braak 1986), and individual inertia is equivalent to the amount of variance that is related solely to the specific variable (the exclusive fraction) or group of variables, after accounting for the variance explained by other variables (the shared fraction) and the interaction between the different variables (Cushman and Mcgarigal 2002). Due to data limitations, we omitted the finest scale from the analysis of mammals in Australia and the two finest scales from the analysis of mammals in the contiguous USA (Tables 2 and 3).

**Table 1:** A description including variable name and data source of all environmental variables used in the analyses of breeding birds and mammals in the contiguous USA and Australia

Variable name	Description	Source
Temperature Temperature seasonality	Standard deviation of monthly temperature values	
Precipitation Precipitation seasonality	Coefficient of variation of monthly precipitation values	Worldclim (Hijmans et al. 2005)
Altitude Altitude range		
NDVI		MODIS – http://glef.uniacs.umd.edu/data/ndvi
Pop-density	Population density	FAOGeoNetwork
Urban*	Urban area	
Forestry*	Forest	
Open-herbaceous*	Herbaceous vegetation	
Agriculture*	Agricultural area	
Water*	Large water body	http://www.fao.org/geonetwork/em/mainhome
Barren*	Dry low vegetation area	
Shrubland*		
Wetland*	Wetland area	
Distance to Urban	Distance to nearest urban area calculated at a fine resolution (0.0083°) and averaged for each grid cell	Data were extracted from ESRI data files

Variables marked “*” represent individual land-use category derived from a single layer containing all other categories marked by “*”.

**Table 2:** Grain size and extent of the 11 scales in the analyses, and the number of sampling grids used for each taxonomic group (birds and mammals) in each study area (contiguous USA and Australia). All scales with valid sampling grids were used in the analyses

Scale	Grain size (km^2^)	Extent (km^2^)	Number of sampling grids in analyses			

			USA mammals	USA breeding birds	Australian mammals	Australian breeding birds
1	10	10,240	–	1237	–	1004
2	20	20,480	–	646	238	529
3	40	40,960	309	334	160	274
4	80	81,920	175	184	107	148
5	160	163,840	97	100	68	78
6	320	327,680	53	56	40	42
7	640	655,360	30	30	22	23
8	1280	1,310,720	17	17	13	13
9	2560	2,621,440	13	14	6	6
10	5120	5,242,880	5	5	5	5
11	10,240	10,485,760	2	2	2	2

**Table 3:** Mean (±SD) number of species of birds and mammals in sampling grids (per spatial scale) in the two study areas. Bottom row shows all species in the study area

Spatial scale	US birds	US mammals	AU birds	AU mammals
1	196.73 (76.75)	23.55 (10.89)	95.31 (41.1)	–
2	234 (64.65)	29.32 (12.68)	112.56 (40.73)	34.71 (18.68)
3	270.98 (61.59)	35.88 (14.99)	168.90 (61.69)	38.58 (19.44)
4	302.02 (66.5)	43.68 (17.47)	191.79 (63.64)	44.17 (21.55)
5	338.4 (63.73)	52.45 (21.58)	216.48 (67.96)	51.67 (23.77)
6	369.6 (76.25)	63.49 (26.42)	246.09 (72.42)	64.8 (28.37)
7	414.1 (78.56)	74.86 (33.14)	272.86 (78.96)	81.09 (32.43)
8	464.82 (66.62)	90.11 (36.1)	307.38 (85.53)	105 (39.34)
9	436.64 (140.65)	93 (47.23)	365.5 (79.64)	147 (39.85)
10	572.6 (82.33)	130.8 (78.76)	396.2 (91.88)	174.2 (54.04)
11	711.5 (19.09)	206 (83.43)	505.5 (50.2)	269.5 (12.02)
Total in study area	804	284	572	371

### Effect of variability in the explanatory variables

The explanatory power of the environmental variables might be correlated with the range of conditions in the sampling grids, that is, the effect per unit change might be constant. In such cases, the differences in explained variance in species composition among different explanatory variables are attributed to the range of variable values (see e.g., Steinitz et al. 2006). Alternatively, the strength of the relationship may also be determined by the ecological affinity of species to the environmental variables, thus even variables with little variability will have strong explanatory power. We examined the per unit effect of climate and LULC variable groups (which consistently explained most of the variance in species composition) by calculating the coefficient of variation (CV) of each variable in each sampling grid used in the analyses. We then fitted a linear regression model to test what amount of explained variance in species composition for each of the species groups at each spatial scale can be attributed to CV. Linear regressions were carried out using R version 2.15.2 (R Core Team 2010). CV was calculated as the ratio of variance of an environmental variable in the sampling grid and the mean value of that variable in that grid. This was possible as the size of the smallest sampling grid cell was coarser than the resolution of the original environmental data layers. A strong correspondence between the amount of explained variance in species composition and the CV of an environmental variable would suggest that the per unit effect is constant and the differences are a result of the amount of variance in the explanatory variable. However, a large amount of explained variance in species composition attributed to environmental variables with low variability will indicate a strong ecological affinity.

## Results

**Figure 1:**
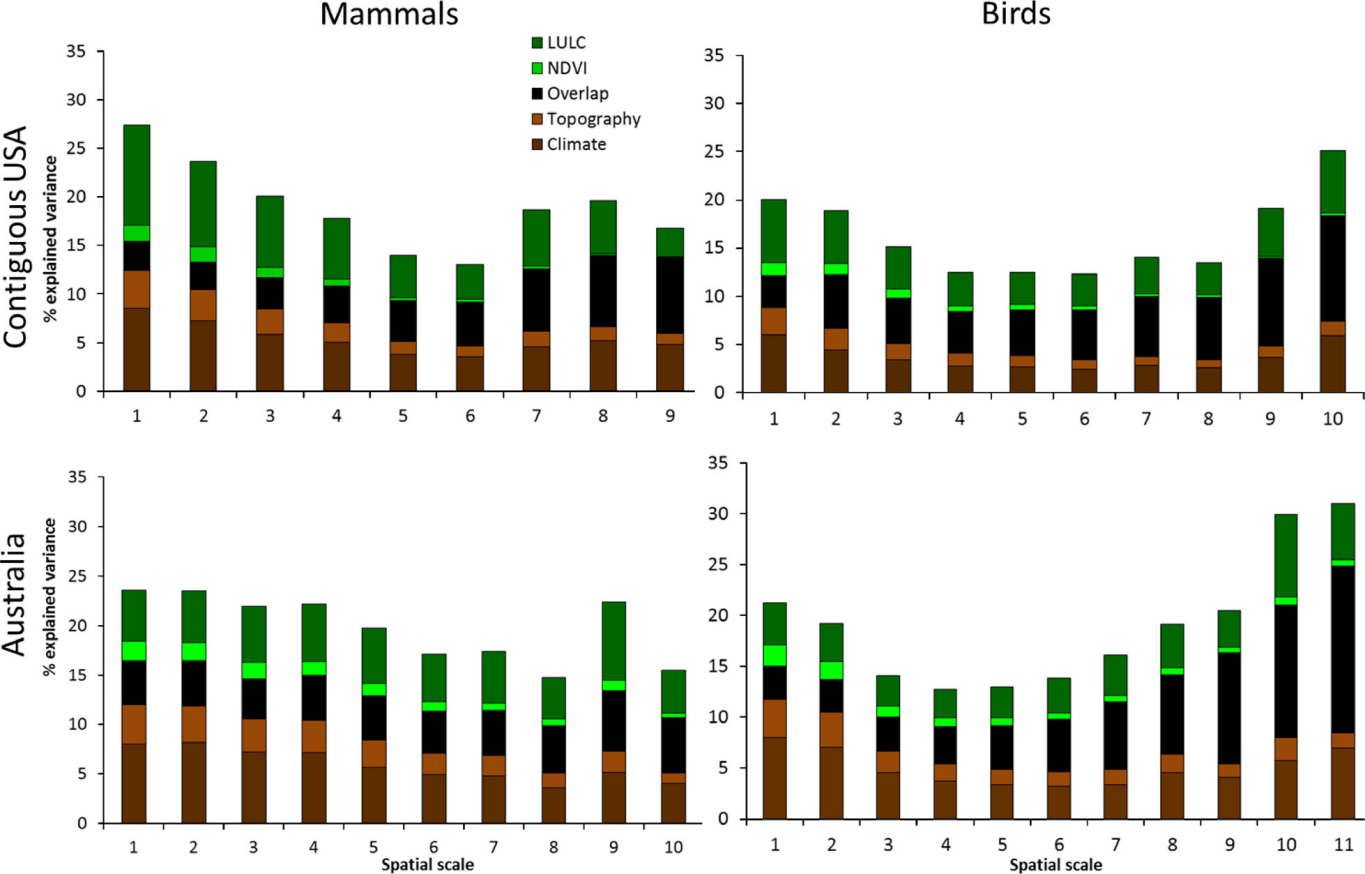
Overall explained variance in mammal and breeding bird species composition in the contiguous USA and Australia. The bars consist of the contribution of climate (dark brown); land use/land cover (LULC) (dark green); topography (light brown); NDVI (light green); and the overlap to overall explained variance (black).

**Figure 2:**
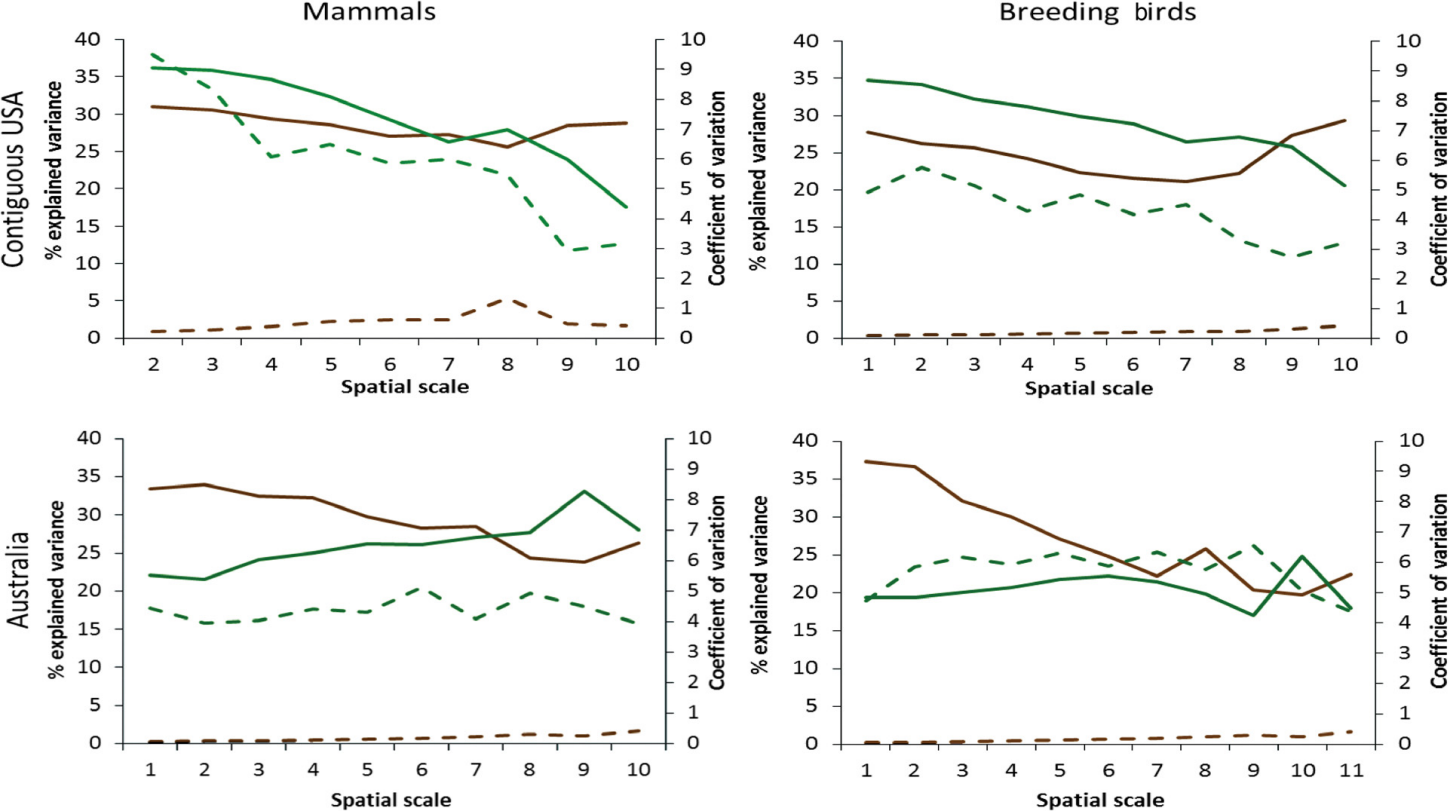
Percent explained variance in mammal and breeding bird species composition in the contiguous USA and Australia, accounted for by climate variables (brown lines) and LULC variables (green lines), as a function of spatial scale of the analysis (full lines). Secondary *y*-axis is the coefficient of variation in climate variables (dashed lines).

Perhaps the only feature common to all four analyses (birds and mammals in Australia and USA) is the finding that most of the explained variance in species composition, at all scales, could be attributed to the combined effect of climate and LULC variables (between 20% and 40% of the total explained variance). Topography and NDVI variables explained a small part of the variation in species composition (<15% and 10%, respectively, Fig. 1). For both topography and NDVI, a similar trend of consistent decline in their importance as scales become coarser was obvious for both taxa in both continents (Fig. 1). In contrast, the effects of climate and LULC were less consistent across scales and varied between taxa and continents. Figure 2 illustrates the overall explained variance, and the unique contribution of climate and LULC variables to it, for taxonomic groups in both continents. Our results are based on the occurrence records of relatively many species, even at the finer scales, suggesting that the finest scale used in this analyses is appropriate for the models we used (Table 3).

In the USA, LULC variables explained more variance than climate at the six and eight finer scales, for mammals and birds, respectively. At coarser scales, climate variables became the strongest explanatory group, and the importance of LULC variables declined (Fig. 2). In contrast, in Australia, a nearly opposite pattern was found, where climate was more important than LULC at fine scales (scales 2–8 in mammals and 1–9 for mammals and birds, respectively), and at coarse scales, both groups had generally similar impact on species composition (Fig. 2).

### The effect of variability in the explanatory variables

The CV of climate always increased with increasing spatial scale, except for a decrease at the coarsest scales for USA mammals (Fig. 2). In contrast, the CV of LULC variables in the USA decreased with scale, while in Australia, there was no obvious trend related to spatial scale (Fig. 2B). The CV of LULC was consistently higher than CV of climate by an order of magnitude (Fig. 2).

We found a major and consistent difference between climate and LULC, in the relationships between the CV of a variable and its relative impact on species composition. The contribution of climate to overall explained variance in species composition, regardless of taxonomic group and geographic location, was significantly and negatively correlated with CV in all combinations except breeding birds in the USA. Mammals in the USA had a regression coefficient of −1.55, *R*^2^_(df = 1,7)_ = 0.8, *P* < 0.001; breeding birds and mammals in Australia had coefficient = −5.03, *R*^2^_(df = 1,9)_ = 0.66, *P* < 0.01; and coefficient = −3.16, *R*^2^_(df = 1,8)_ = 0.73, *P* = 0.01, respectively (Fig. 2A). There was a strong positive correspondence between CV of LULC variables and their contribution to the overall explained variance in both taxonomic groups in the USA. Birds had regression coefficient of 3.47, *R*^2^_(df = 1,8)_ = 0.65, *P* = 0.005, while mammals had coefficient = 5.45, *R*^2^_(df = 1,7)_ = 0.77, *P* = 0.001. (Fig. 2B). In Australia, CV of LULC had no significant effect on either taxonomic group's explained variance in species composition.

## Discussion

Our analyses of occurrence records of birds and mammals in two distant continents confirmed that the effect of environmental variables on species composition is scale-dependent and that climate and LULC are the major environmental factors affecting species composition of these two taxonomic groups consistently. Topography and primary productivity probably have a noticeable impact on species composition at fine scales that diminishes at coarser scales. Unfortunately, there is still a lack of high-resolution data for large geographical extents to allow such analyses.

Attempts have been made to find a way to empirically calculate the ‘correct’ scale of analysis for specific ecological systems (Keeling et al. 1997; Pascual and Levin 1999; Habeeb et al. 2005). Our results corroborate the notion that analyzing data at multiple spatial scales in a single study, in order to capture processes affecting studied patterns, is superior to any single-scale analysis (Fortin and Dale 1999; Cushman and Mcgarigal 2002; Grand and Cushman 2003; Willig et al. 2003; Kent et al. 2011). Multiscale studies have been used to characterize relationships between species composition and the environment (Grand and Cushman 2003; Cushman and Mcgarigal 2004), but most studies used a qualitative definition of scale, and their range of scales was narrower than the range of scales in this study. The grains used in this study are much finer compared with grains used in continental scale analyses. For example, Dobson et al. (1997) used counties and states to analyze endangered species in the USA; Bickford and Laffan (2006) used a grid of 50 × 50 km to correlate species richness of pteridophytes and climate; Grenyer et al. (2006) used a 96.3 × 96.3 km grid in their analyses of rare threatened vertebrates. The present study used a minimal grain size of 10 km^2^, at least 10,000 times smaller than these studies.

The use of multiple spatial scales revealed the most striking result of this study, which is the contrasting and consistent difference between the effect of climate and the effect of land use–land cover on species composition (Fig. 2). These two groups of variables had the largest effect on species composition at all studied scales for both mammals and birds, in the USA as well as in Australia. In all these cases, the impact of LULC variables was positively related to the variance in these environmental variables. In contrast, the impact of climatic variables changed with scale irrespective of their variance, and in some cases even in contrast to patterns of variability in the explanatory variables, which was consistently small.

We tested an approach that may render an ecological perspective to these findings. We hypothesized that the impact of a certain explanatory variable on species distribution is a product of two elements: the net ecological affinity of the species to this variable and its variance. Hence, a strong impact of an environmental variable indicates either strong affinity to it or that it has large variance. If both conditions are met, then the observed impact is expected to be very strong. In our analyses, there was a close correspondence between the coefficient of variation in LULC variables and the variance in species composition explained by LULC, suggesting that the strength of the effect of LULC on species composition is largely attributed to the variability in LULC variables. The relatively large proportion of variance explained by climatic variables, despite the relatively low variability in climate on both continents (an order of magnitude lower than in LULC), suggests a stronger affinity of birds and mammals to climatic conditions, compared with their affinity to LULC. Our results indicate that these two major environmental variable groups, largely accepted as drivers of biodiversity distribution patterns, operate in distinct manners to affect species composition patterns. This difference is consistent in both mammals and birds, as well as among distant continents.

Breaking down the overall explained variance to assess the specific effect of each variable group revealed that in all four analyses (mammals in both continents and breeding birds in both continents), the patterns of climatic-related explained variance were similar. This coincides with high ecological affinity and low spatial variability, as measured in our samples. In contrast, we found a substantial difference between the amounts of variance in composition of both groups explained by LULC variables between the two continents. As the amount of explained variance was positively correlated with the coefficient of variation in LULC variables, it is reasonable to relate the differences between continents to the different spatial patterns of LULC. While in Australia, most variability in LULC patterns is concentrated along the coastal regions, and most of the inland areas are either desert or farmland, LULC patterns in the continental USA are more diverse. Another aspect of the interaction between variability in environmental conditions and species composition may be related to the location of the assemblage along the major environmental gradient (Steinitz et al. 2006). Such analyses would require a sampling design that will allow to isolate the effect of the location of the assemblage along the major gradient from other factors, such as variability and inherent ecological affinity, and is thus beyond the scope of this study. In addition, environmental gradients may account for only a certain fraction of the variance in spatio-temporal species composition patterns, while the remaining variance is perhaps accounted for by neutral processes related to dispersal limitations and species' demographic characteristics (Hubbell 1997, 2001) and to interspecific interactions (Wilson et al. 2003).

Our results demonstrate that both bird and mammal species composition is influenced by environmental factors either directly affected by humans, such as LULC variables, or indirectly affected by them, such as climate. It is reasonable to conclude, in light of these results, that ongoing environmental change, as predicted by the report of the Intergovernmental Panel on Climate Change, will lead to significant global changes in species composition at all scales. The realization that there is more than a single mechanism affecting species-environment relationships is an important step toward understanding them, which might eventually help in predicting the effects of global change on biodiversity.

## Appendix

The issue of spatial scale in ecological studies has been the subject much debate (Allen & Hoekstra, 1992; Wiens, 1989; Schneider, 2001). It has become unequivocal that scale plays an important role in the effect environmental conditions have on ecological states and processes, and thus on patterns of biodiversity distribution. Due to the complex nature of scale, it is not trivial to evaluate its impact, disentangling it from other factors. In particular, scale consists of two major elements, grain and extent, and a change of scale may be a change in grain, or in the extent, or both. This complexity was rarely treated explicitly in previous efforts to study the impact of scale.

Here we used a systematic upscaling approach, in which we kept a constant ratio between grain and extent, in order to alter both the lengths of environmental gradients, and the basic ecological unit of the analysis. This approach holds a principal advantage over exclusively altering either one of the components of scale. Some environmental variables are heterogeneous at small scales (i.e. change over short geographical distances, such as soil type for example) while others vary over large geographical distances (e.g. climatic variables). While altering a single component of scale may detect patterns of either small or large scale variables, it will probably fail in detecting the opposite. Moreover, some variables may change over intermediate distances, and some may be heterogeneous at multiple scales. Thus, only a systematic alteration of both components of scale may detect the effect of variables at all levels of heterogeneity. Furthermore, by using an unbalanced rescaling scheme, one is expected to receive partially erroneous results, leading to inaccurate conclusions, as patterns are expected to deviate from their true form (Kent and Carmel, unpublished data).
